# Synthesis of 3D Hollow Layered Double Hydroxide-Molybdenum Disulfide Hybrid Materials and Their Application in Flame Retardant Thermoplastic Polyurethane

**DOI:** 10.3390/polym14081506

**Published:** 2022-04-07

**Authors:** Yi Qian, Wenyuan Su, Long Li, Haoyan Fu, Jiayin Li, Yihao Zhang

**Affiliations:** 1 College of Chemical Engineering, Qingdao University of Science and Technology, Qingdao 266042, China; suwenyuan1226@163.com (W.S.); fuhaoyanfhy@163.com (H.F.); 2 College of Environment and Safety Engineering, Qingdao University of Science and Technology, Qingdao 266042, China; lijiayin085@163.com (J.L.); 18832623721@163.com (Y.Z.)

**Keywords:** layered double hydroxide, molybdenum disulfide, thermoplastic polyurethane, flame retardant

## Abstract

The development of high-performance thermoplastic polyurethane (TPU) with high flame retardancy and low toxicity has always been the focus of its research. In this paper, the novel 3D hollow layered double hydroxide/molybdenum disulfide (LDH/MoS_2_) hybrid materials were synthesized by hydrothermal method using the MIL-88A as in situ sacrificial template and MoS_2_ as synergistic flame retardant. Among all TPU composites, the peak heat release rate, total heat release rate, and total smoke release rate of TPU/NiFeTb-LDH/MoS_2_ were reduced by 50.9%, 18.2%, and 35.8% compared with pure TPU, respectively. The results of the thermogravimetric infrared analysis demonstrated that the contents of combustible volatiles (hydrocarbons) and toxic volatiles (CO and HCN) emitted from TPU/LDH/MoS_2_ were significantly reduced, indicating that LDH/MoS_2_ hybrid materials can dramatically enhance the fire safety of TPU composites. Combined with the analysis of carbon residues and thermal stability of TPU composites, the enhanced flame retardancy and smoke suppression performances are primarily attributed to the catalytic carbonization of LDH and the physical barrier effect of MoS_2_.

## 1. Introduction

Thermoplastic polyurethane (TPU) is widely used in various fields such as food, medical, clothing, cables, automobiles, etc. owing to its excellent wear resistance, high stability, and mechanical performance [1,2,3]. Nevertheless, similar to most polymers, TPU is highly flammable and emits a great number of toxic gases and fumes during combustion, which dramatically limits its applications [4]. Consequently, flame retardant modifications of TPU composites are essential, especially in some fields with high requirements for fire resistance. Traditional halogen flame retardants have the advantages of high flame retardant efficiency, low addition amount and low cost. However, due to the generation of harmful gases such as dioxins during the combustion process, halogen flame retardants have been reduced or even banned in some areas [5]. In recent years, some new halogen-free flame retardants have been proved to improve the fire resistance and thermal stability of polymer, such as layered double hydroxide (LDH) [6], molybdenum disulfide (MoS_2_) [7], graphene oxide (GO) [8], etc.

Layered double hydroxide (LDH), also known as anionic clay, is a lamellar nanomaterial composed of positively charged layers and interlayer anions [9]. LDH has a wide range of applications in flame retardancy, adsorption and catalysis due to its high thermal stability, tunable chemical composition and high anion exchange capacity [10]. LDH endows polymer with good smoke suppression and flame retardancy properties mainly by generating protective carbon layers and diluting combustible gases during combustion.

It is well known that the flame retardancy of LDH is closely related to its good dispersion in polymer. Nonetheless, inorganic LDH is not well dispersed in hydrophobic polymer due to its hydrophilic surface [11]. Hence, it is necessary to develop a novel LDH that can make full use of the advantages of two-dimensional (2D) LDH while avoiding the self-stacking of 2D LDH. In recent years, the use of metal-organic frameworks (MOFs) as self-sacrificial templates to construct three-dimensional (3D) hollow LDH has attracted extensive attention. MOFs are porous, contain metal and organic components, and have weak physical and chemical stability, making MOFs particularly suitable for precursors or template materials [12]. After the unstable core of MOFs is etched, ultrathin LDH nanosheets are obtained by in-situ transformation and deposition, thus effectively avoiding the self-stacking of LDH [13]. In addition, the cavity can release the metal sites deeply hidden in the MOFs framework, which can effectively improve the utilization of metal sites. However, the only MOFs reported as precursors are ZIF-67, ZIF-8, and MIL-88A. For instance, Zhou et al. [14] synthesized 3D NiCo-LDH@PZS hollow dodecahedral structure using ZIF-67 as a precursor and in situ sacrificial template, and introduced it into epoxy resin (EP). The results showed that the peak heat release rate (PHRR) and the total heat release (THR) of EP/NiCo-LDH@PZS are reduced by 30.9% and 11.2%, respectively, compared with pure EP.

As a typical representative of layered metal sulfides, MoS_2_ is a more commercially valuable 2D layered material with a similar structure to graphene [15]. Transition metal element Mo can promote the formation of carbon layer in the polymer matrix, thus improving the flame retardancy and smoke suppression properties of the polymer [16]. Compared with graphene, MoS_2_ has lower thermal conductivity, which is not conducive to heat transport within the polymer matrix and thus can effectively delay thermal degradation [17]. However, the exfoliated MoS_2_ nanosheets are easily aggregated in the polymer matrix due to the van der Waals force, so the satisfactory flame retardancy effect cannot be achieved by MoS_2_ alone [18]. It has been reported that the compounding of MoS_2_ with other conventional flame retardants can not only prevent the agglomeration of MoS_2_ but also improve the thermal stability and flame retardancy of the polymer [16]. Till now, there are few studies on 3D hollow LDH/MoS_2_ hybrid materials as flame retardant.

In this paper, rare earth ions (Ce^3+^/Tb^3+^) doped MIL-88A were synthesized. The introduction of Ce^3+^/Tb^3 +^ can protect the outer structure of MIL-88A from the attack of alkaline media, while the inner structure with weak stability was selectively eroded under alkaline conditions, novel 3D hollow LDH was obtained. In addition, then 3D hollow LDH was hybridized with MoS_2_ nanosheets to construct 3D hollow LDH/MoS_2_ hybrid materials. and employed as nanofillers for TPU. The cone calorimeter and thermogravimetric-infrared results demonstrated that the flame retardancy and smoke suppression performances, thermal stability, and fire safety of TPU/LDH/MoS_2_ were significantly enhanced. Combined with the carbon residues analysis of TPU composites, the enhanced flame retardancy properties are mainly attributed to the catalytic carbonization and physical barrier effects of LDH/MoS_2_ hybrid materials.

## 2. Experimental

### 2.1. Materials

Iron (III) nitrate nonahydrate (Fe(NO_3_)_3_·9H_2_O, AR) was supplied by Aladdin Chemical Reagent Manufacturing Co. Ltd., Ontario, CA, USA. Cerium (III) nitrate hexahydrate (Ce(NO_3_)_3_·6H_2_O, AR), urea (CH_4_N_2_O, AR) and fumaric acid (C_4_H_4_O_4_, AR) were bought from McLean Chemical Reagent Co. Ltd., London, UK. Nickel (II) nitrate hexahydrate (Ni(NO_3_)_2_·6H_2_O, AR) were supplied by Tianjin Dingshengxin Chemical Co. Ltd., Tianjin, China. Molybdenum disulfide (MoS_2_) and n-butyllithium (C_4_H_9_Li, AR) were supplied by Sinopharm Group Chemical Reagent Co. Ltd., Shanghai, China. Thermoplastic polyurethane (TPU, 9380A) was purchased from Germany’s bayer. Deionized water was used during the experiments.

### 2.2. Synthesis of 3D Hollow NiFeCe-LDH and NiFeTb-LDH

The 3D hollow NiFeCe-LDH and NiFeTb-LDH were synthesized according to previous literature with modification [19]. Firstly, 0.348 g of fumaric acid and 1.44 g of urea were added into 60 mL of deionized water followed by stirring for 30 min at room temperature. Then Fe(NO_3_)_3_·9H_2_O, Ce(NO_3_)_3_·6H_2_O and Ni(NO_3_)_2_·6H_2_O (2.835 g) were successively added to the above solution, and the total amount of Ce(NO_3_)_3_·6H_2_O and Fe(NO_3_)_3_·9H_2_O is fixed at 3.25 mM. Then, the completely dissolved mixture was poured to the 100 mL Teflonlined autoclave and reacted at 110 °C for 12 h. After the reaction, it was cooled to room temperature, and the obtained product was washed with deionized water and ethanol for three times. The washed precipitate was dried in a vacuum drying oven at 60 °C for 12 h to obtain 3D hollow NiFeCe-LDH and NiFeTb-LDH.

### 2.3. Synthesis of 3D Hollow NiFeCe-LDH/MoS_2_ and NiFeTb-LDH/MoS_2_ Hybrid Materials

**Figure 1:**
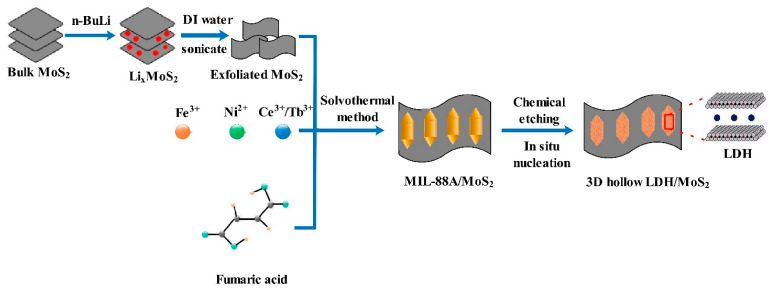
Schematic Diagram of 3D hollow LDH/MoS_2_.

The exfoliated MoS_2_ nanosheets were prepared according to the previously reported method [20]. In addition, 3D hollow NiFeCe-LDH/MoS_2_ and NiFeTb-LDH/MoS_2_ hybrid materials were synthesized under the comparable experimental conditions as above. The mass fraction of MoS_2_ in the obtained hybrid materials was fixed to 3 wt%. Figure 1 is the schematic diagram of the preparation process of the 3D hollow LDH/MoS_2_ hybrid materials.

### 2.4. Synthesis of TPU Composites

Preparation of TPU composites via melt blending method. Under the conditions of an internal mixing temperature of 180 °C and rotating speed of 30 rpm, TPU and the prepared flame retardants (the additional amount of flame retardants is 2 wt%) were mixed in an internal mixer in a certain proportion until they are completely mixed. Then, the TPU composites were hot-pressed for 10 min and cold-pressed for 3 min at 180 °C and 10 MPa. Finally, the pressed TPU composites were cut to the appropriate size (100 × 100 × 3 mm^3^) for subsequent testing.

### 2.5. Characterization

X-ray diffraction (XRD) patterns were conducted on a Philips X’Pert Panalytical diffractometer using Cu-Kα radiation (λ = 0.1542 nm). Fourier transform infrared spectroscopy (FTIR) spectra were made with an IRAfnity-1 FTIR spectrophotometer (PerkinElmer, Waltham, MA, USA) with a test range of 400–4000 cm^−1^. X-ray Photoelectron Spectroscopy (XPS) performed by AXIS SUPRA Spectrometer (Kratos, London, UK). The Brunauer-Emmett-Teller (BET) specific surface area was measured by N_2_ adsorption-desorption method on an ASAP2020 system. Scanning electron microscope (SEM) images were acquired using a JSM-6700F instrument with a parameter condition of 5 kV. Transmission Electron Microscopy-Energy Dispersion (TEM-EDS) measurements were performed using a JEOL-2010 instrument. The TPU composites were determined by the JCZ-2 cone calorimeter according to ISO 5660 standard. The TPU composites with a size of 100 × 100 × 3 mm^3^ were wrapped in aluminum foil, placed horizontally on the sample rack and heated with an external heat source of 50 kW/m^2^. Thermogravimetric analysis-infrared spectrometry (TG-FTIR) was performed on a DT-50 instrument at a heating rate of 20 °C/min (N_2_ atmosphere), and the temperature range was 40–800 °C.

## 3. Results and Discussion

### 3.1. Characterization of 3D Hollow LDH and Its Hybrid Materials

**Figure 2:**
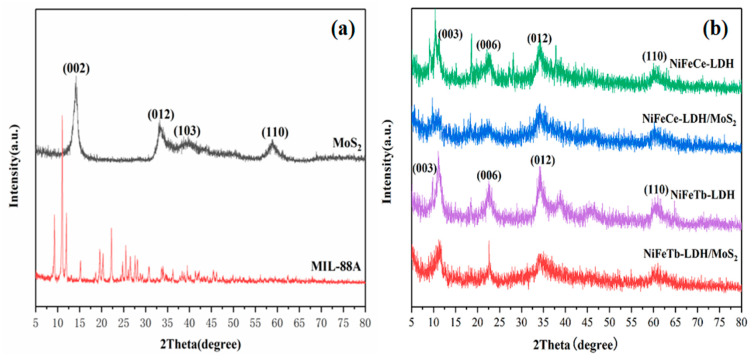
XRD patterns of MIL-88A, MoS_2_ (**a**) and NiFeCe-LDH, NiFeTb-LDH, NiFeCe-LDH/MoS_2_, NiFeTb-LDH/MoS_2_ (**b**).

The crystallinity of MIL-88A, MoS_2_, MIL-88A derived LDH and LDH/MoS_2_ hybrid materials were investigated by XRD, as shown in Figure 2. It can be seen from Figure 2a that the diffraction peak of exfoliated MoS_2_ at 2θ = 14.2° corresponds to the (002) plane [18]. The diffraction peaks of the as-synthesized MIL-88A are in good agreement with the results reported in the previous literature [21]. The (003), (006), (012) and (110) planes occurring in LDH correspond to the typical characteristic peaks of LDH, indicating that NiFeCe-LDH and NiFeTb-LDH have been successfully synthesized [19]. Meanwhile, there are no diffraction peaks corresponding to MIL-88A in the obtained LDH materials, indicating that MIL-88A is completely transformed into LDH. It is worth noting that NiFeCe-LDH and NiFeTb-LDH have broad diffraction peaks, which may be due to the fact that the ionic radii of Ce^3+^ (102 pm) and Tb^3+^ (92.3 pm) are much larger than that of Fe^3+^ (55 pm), causing lattice distortion of LDH. The XRD patterns of LDH/MoS_2_ and LDH have similar peaks, corresponding to the characteristic peaks of LDH. This is mainly due to the fact that the LDH nanosheets grown on the surface of MoS_2_ destroy the face-to-face stacking structure of MoS_2_ nanosheets, which can effectively inhibit the restacking of the MoS_2_ nanosheets [22].

**Figure 3:**
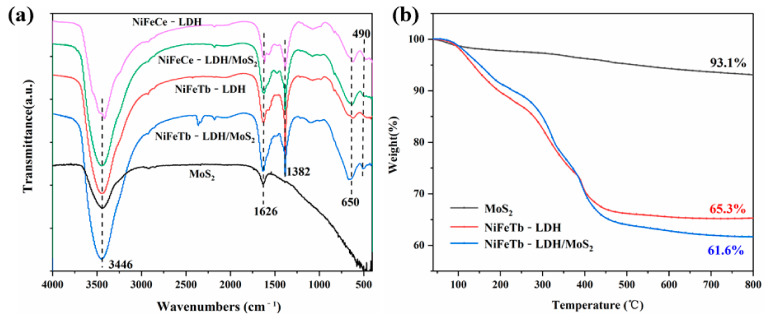
FTIR spectra of MoS_2_, NiFeCe-LDH, NiFeCe-LDH/MoS_2_, NiFeTb-LDH and NiFeTb-LDH/MoS_2_ (**a**); TG curves of MoS_2_, NiFeTb-LDH and NiFeTb-LDH/MoS_2_ (**b**).

The FTIR spectra of NiFeCe-LDH/MoS_2_ and NiFeTb-LDH/MoS_2_ hybrid materials together with MoS_2_, NiFeCe-LDH and NiFeTb-LDH are depicted in Figure 3a. It can be seen from Figure 3a that all five materials show absorption peaks at 3446 cm^−1^ and 1626 cm^−1^, which are related to the stretching vibration of the -OH group and the bending vibration of the water molecules in the middle layer. As for NiFeCe-LDH and NiFeTb-LDH, the positions of their absorption peaks are basically the same. For instance, the absorption peak at 1382 cm^−1^ is attributed to the stretching vibration of NO_3_^−^ as interlayer anion, and the absorption peaks below 700 cm^−1^ correspond to the stretching vibration of metal-OH in the LDH structure, which further proves that the prepared materials are metal hydroxides [23]. In addition, it is worth noting that the positions of the absorption peaks of NiFeCe-LDH/MoS_2_ and NiFeTb-LDH/MoS_2_ are similar with those of NiFeCe-LDH and NiFeTb-LDH, which is consistent with the analysis results of XRD. The thermal decomposition behaviors of MoS_2_, NiFeTb-LDH, and NiFeTb-LDH/MoS_2_ under N_2_ atmosphere were investigated by TGA, and the TG results are shown in Figure 3b. It can be seen from Figure 3b that the MoS_2_ nanosheets have high thermal stability with a mass loss of only 6.9%. In contrast, NiFeTb-LDH presents three decomposition stages: the first mass loss stage of NiFeTb-LDH occurs in the range of 50 °C and 200 °C corresponding to the loss of interlayer water; the second mass loss stage appears between 250 °C and 370 °C, which is related to the decomposition of metal hydroxides; the third mass loss stage occurs between 450 °C and 550 °C, which can be attributed to the collapse of metal organic frameworks [24]. At 800 °C, the char yield of NiFeTb-LDH is 65.3%. For NiFeTb-LDH/MoS_2_, it has a similar thermal decomposition trend to NiFeTb-LDH, which might be ascribed to the higher content of NiFeTb-LDH in the NiFeTb-LDH/MoS_2_ hybrid material. The char yield of NiFeTb-LDH/MoS_2_ at 800 °C is 61.6%, which is only slightly lower than that of NiFeTb-LDH.

**Figure 4:**
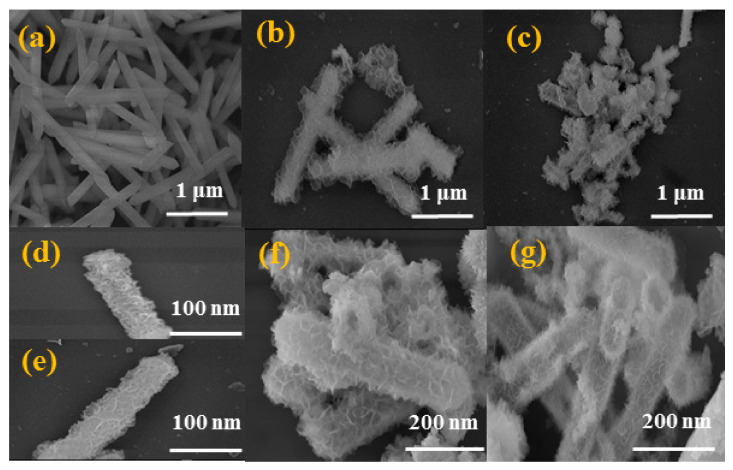
SEM images of MIL-88A (**a**), NiFeCe-LDH (**b**,**d**), NiFeTb-LDH (**c**,**e**), NiFeCe-LDH/MoS_2_ (**f**) and NiFeTb-LDH/MoS_2_ (**g**).

The morphologies of MIL-88A, NiFeCe-LDH, NiFeTb-LDH, NiFeCe-LDH/MoS_2_ and NiFeTb-LDH/MoS_2_ were characterized by SEM. It can be observed from Figure 4a that the precursor MIL-88A presents a hexagonal micro rod structure with a uniform size (about 2 μm in length and 500 nm in width) and a smooth surface. As revealed in Figure 4b–e, NiFeCe-LDH and NiFeTb-LDH still maintain the hexagonal microrod structure of precursor, but their surfaces are rougher, indicating that LDH nanosheets are successfully synthesized on the surface of nano frame. As shown in Figure 4f,g, is a failure to find MoS_2_ nanosheets in the SEM images of NiFe-Ce-LDH/MoS_2_ and NiFeTb-LDH/MoS_2_ hybrid materials, which may be caused by the exfoliated MoS_2_ nanosheets completely covered by LDH [25]. In order to investigate the distribution of elements, plan scan image and EDS spectrum analysis were performed on NiFeCe-LDH/MoS_2_ and NiFeTb-LDH/MoS_2_ hybrid materials. As shown in Figure S1a,c, we can clearly see that Fe, Ni, and Ce/Tb elements are uniformly distributed throughout the nano frame, which proves that the composition of LDH is ternarily doped. Meanwhile, the detected Mo elements also corroborate the presence of MoS_2_, indicating the successful preparation of LDH/MoS_2_ hybrid materials.

**Figure 5:**
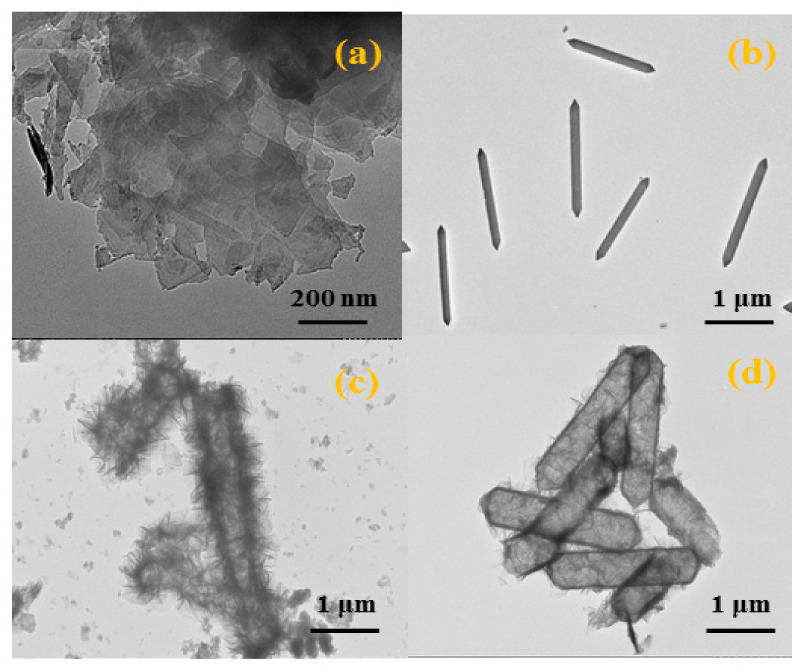
TEM images of MoS_2_ (**a**), MIL-88A (**b**), NiFeCe-LDH (**c**), NiFeTb-LDH (**d**).

The structural features of MoS_2_, MIL-88A, NiFeCe-LDH, and NiFeTb-LDH were further characterized by TEM. It can be seen from Figure 5a that the exfoliated MoS_2_ nanosheets present typical two-dimensional sheet-like morphology with a size of about 200 nm. However, MoS_2_ nanosheets exhibit different degrees of restacking in some areas due to van der Waals forces. From Figure 5b, we can observe that the precursor MIL-88A is a solid structure. As shown in Figure 5c,d, NiFeCe-LDH and NiFeTb-LDH possess distinct hollow structures with an average length of about 1 μm. The ultrathin and uniform LDH nanosheets are mainly inserted vertically on the surface of the hollow nano frame, which effectively inhibits the aggregation of LDH nanosheets. In addition, the loose stacking of LDH nanosheets on the precursor surface forms a highly porous structure, thus NiFeCe-LDH and NiFeTb-LDH have high specific surface areas of 98.2047 m^2^/g and 100.7177 m^2^/g, respectively (Figure S2).

To further confirm the chemical composition of LDH/MoS_2_ hybrid materials, the XPS characterization was carried, and the results are shown in Figure S3. XPS results show that LDH/MoS_2_ hybrid materials are composed of C, O, Fe, Ni, Ce/Tb and Mo elements, which also corresponds to the EDS results of LDH/MoS_2_ hybrid materials. The peak signal of Mo is lower due to the small amount of MoS_2_ added in the LDH/MoS_2_ hybrid materials. In the high-resolution Fe 2p spectrum, there are two typical peaks located at 712.5 eV and 725.1 eV, which indicate that Fe mainly exists in the positive trivalent state in the LDH/MoS_2_ hybrid materials. Ni, Ce/Tb and Mo perform divalent, trivalent and tetravalent states in LDH/MoS_2_ hybrid materials, respectively.

### 3.2. CCT Tests of TPU Composites

Cone calorimeter based on the principle of oxygen consumption has been widely used to evaluate the combustion performance of materials, which can obtain important physical parameters related to heat and smoke [26]. Table 1 shows the specific data of the cone calorimeter test (CCT).

**Figure 6:**
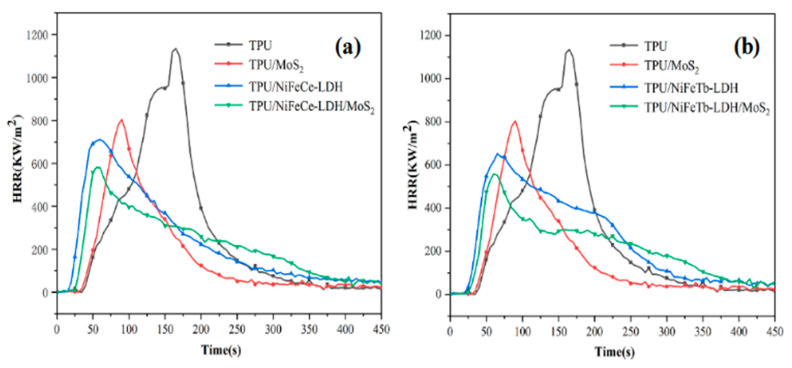
HRR curves of pure TPU and TPU composites (**a,b**).

Heat release rate (HRR) is an important parameter to evaluate the fire risk level of materials. It can be used to predict the size and spread rate of fire [26]. As can be seen from Figure 6, the pure TPU is highly flammable and reaches the peak heat release rate (PHRR) with a value of 1135 kw/m^2^ at around 175 s. Compared with pure TPU, the HRR curves of MoS_2_, NiFeCe-LDH and NiFeTb-LDH filled TPU are relatively flat, and the PHRR decreases from 1135 kW/m^2^ to 804 kW/m^2^, 710 kW/m^2^, and 652 kW/m^2^, corresponding to 29.2%, 37.4%, and 42.6% decrement, respectively. This can be attributed to the fact that the physical layered structure of MoS_2_ and LDH can play a role of sheet barrier during TPU combustion. In addition, the decomposition of LDH at high temperature can release water vapor and absorb heat, thereby reducing the combustion rate of the TPU matrix. It is interesting to note that the PHRR values of TPU filled with NiFeCe-LDH/MoS_2_ and NiFeTb-LDH/MoS_2_ hybrid materials further decreased to 581 kW/m^2^ and 557 kW/m^2^, which decreased by 18.2% and 14.6% compared with that of TPU/NiFeCe-LDH and TPU/NiFeTb-LDH, respectively. This indicates that MoS_2_ and LDH have a good synergistic effect in improving the flame retardancy of TPU. On the one hand, metal oxides formed by thermal decomposition of LDH can promote the formation of a protective carbon layer on TPU matrix, which can prevent the heat transfer of the system and reduce the concentration of combustible gas in the system [27]. On the other hand, MoS_2_ nanosheets have large contact area, which can prevent the penetration of external heat and oxygen, suppress the release of combustible pyrolysis products, and then promote the carbonization of TPU matrix [28]. As can be seen from Figure 6, the ignition time of TPU composites containing MoS_2_ is slightly prolonged than that of TPU composites without MoS_2_, which may be related to the fact that the two-dimensional structure of MoS_2_ nanosheets inhibits the release of combustible gases during the ignition phase.

**Figure 7:**
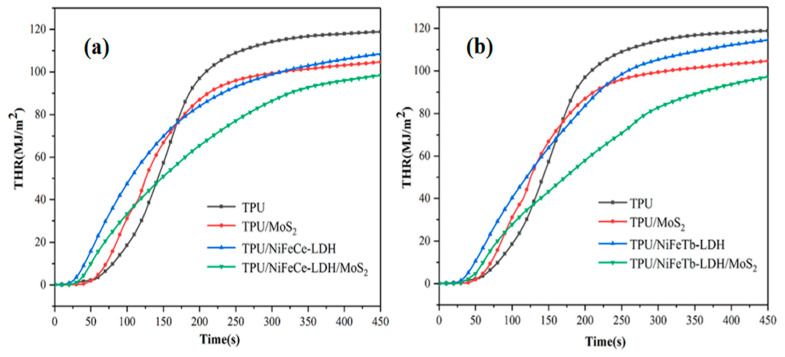
THR curves of pure TPU and TPU composites (**a,b**).

The total heat release (THR) curves of pure TPU and TPU composites are shown in Figure 7. As can be seen from Figure 7, the THR value of pure TPU is the largest, reaching 118.8 MJ/m^2^. Compared with pure TPU, the THR values of TPU/MoS_2_, TPU/NiFeCe-LDH and TPU/NiFeTb-LDH decreased to 104.6 MJ/m^2^, 108.5 MJ/m^2^ and 114.6 MJ/m^2^, respectively. Notably, compared with TPU composites filled with single MoS_2_ or LDH, TPU composites with the introduction of NiFeCe-LDH/MoS_2_ and NiFeTb-LDH/MoS_2_ hybrid materials have lower THR values of 98.4 MJ/m^2^ and 97.2 MJ/m^2^, respectively. This is mainly attributed to the better catalytic carbonization effect of the LDH/MoS_2_ hybrid materials during the combustion process. As a protective barrier, char can prevent the outflow of pyrolysis products from the decomposition zone, so as to slow down the spread of fire and reduce the total heat release [29]. At the same time, the CO_2_ produced by the organic ligand fumaric acid during the combustion process will dilute the combustible gas, which can inhibit the further combustion of the TPU to a certain extent.

**Figure 8:**
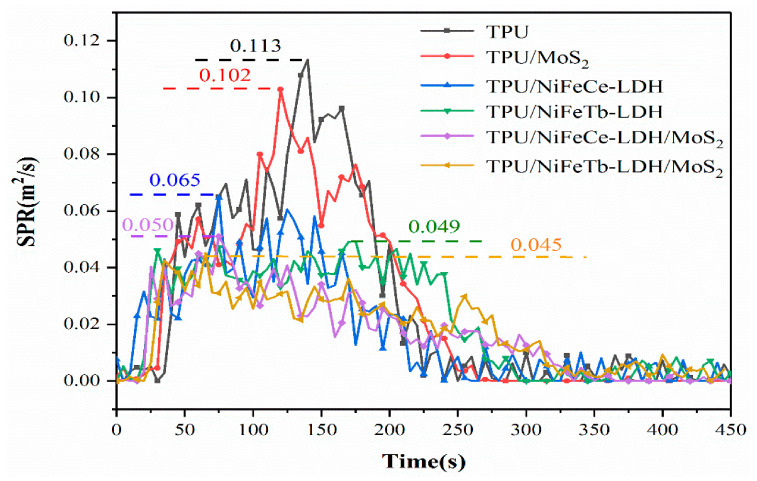
SPR curves of pure TPU and TPU composites.

The hazard of smoke from polymer combustion is another important lethal factor in addition to thermal hazard. Therefore, flame retardants must keep smoke generation to a minimum to reduce fire hazards [14]. The smoke production rate (SPR) curves of pure TPU and TPU composites are illustrated in Figure 8. As can be seen from Figure 8, the peak smoke production rate (PSPR) of pure TPU is as high as 0.113 m^2^/s. Compared with pure TPU, the PSPR value of the TPU/MoS_2_ is only reduced by 9.7%, indicating that the smoke suppression effect of single MoS_2_ is not ideal. In contrast, the PSPR values of TPU/NiFeCe-LDH and TPU/NiFeTb-LDH are lower by 42.5% and 56.7%, respectively. This is mainly explained by metal oxides produced by LDH in the process of thermal degradation can adsorb incompletely burned carbon particles [30]. In the meantime, the water vapor and CO_2_ produced during the thermal decomposition of LDH can dilute part of the flue gas. It can be clearly found that after adding NiFeCe-LDH/MoS_2_ and NiFeTb-LDH/MoS_2_ hybrid materials, the PSPR values of TPU composites are further reduced and the SPR curves are relatively flat, demonstrating that MoS_2_ nanosheets and LDH have a remarkable synergistic smoke suppression effect. On the one hand, MoO_3_ particles produced by the oxidation of MoS_2_ have an efficient smoke suppression effect; on the other hand, the transition metal element Mo plays a catalytic role in TPU composites, and the generated carbon layer can delay the release of smoke particles.

**Figure 9:**
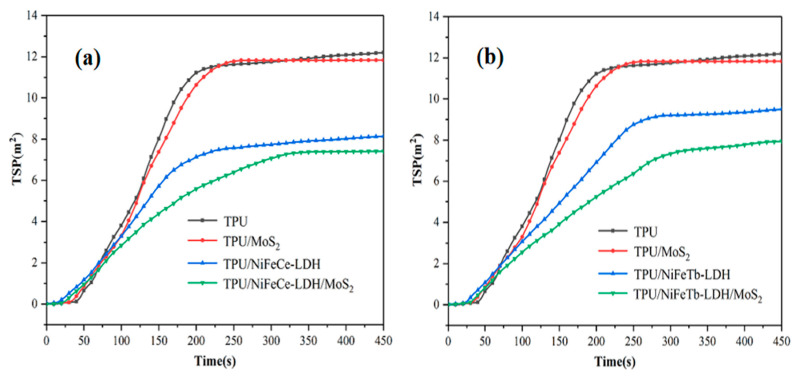
TSP curves of pure TPU and TPU composites (**a,b**).

The total smoke production (TSP) curves of pure TPU and TPU composites are illustrated in Figure 9. It can be observed that pure TPU has the highest TSP with a value of 12.19 m^2^. Nonetheless, the addition of MoS_2_ has little effect on the TSP value, which can be explained by the poor dispersion of MoS_2_ nanosheets due to the existence of the van der Waals force, so that MoS_2_ nanosheets can’t give full play to the nano barrier effect. In comparison with the pure TPU, the TSP values of TPU/NiFeCe-LDH and TPU/NiFeTb-LDH decreased by 33.2% and 22.1%, respectively. This is mainly due to the fact that LDH would help to promote charring. The formation of carbon layer increases the difficulty of escaping combustible, which can further reduce combustible gas and smoke-forming materials in the gas phase [31]. Compared with TPU/MoS_2_ and TPU/LDH, the TSP values of LDH/MoS_2_ filled TPU are further reduced. This is mainly owing to the fact that organic volatiles mainly stay in the condensed phase, which is the main source of smoke particles [32].

### 3.3. Thermal Stability of TPU Composites

**Figure 10:**
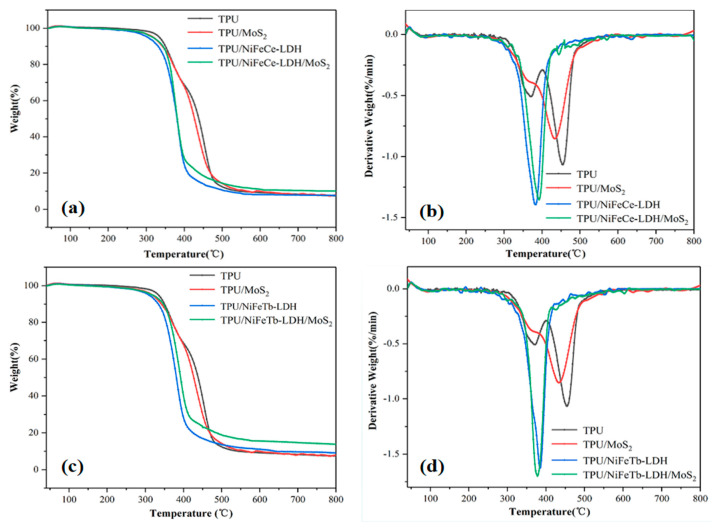
TG (**a,c**)and DTG (**b,d**) curves of TPU and TPU composites.

Based on the fact that the thermal behaviors of TPU composites are closely associated with the flame retardancy, the thermal performances of the TPU composites were evaluated by the thermogravimetric analyzer (TGA) in N_2_ [33]. Figure 10 exhibits the TG and DTG curves of pure TPU and TPU composites, and the detailed data are provided in Table 2. It can be observed that the thermal decomposition behavior of pure TPU is divided into two stages, which is ascribed to the chain scission of carbamates on the TPU backbone and the decomposition of polyols in the soft segment [34]. It can be clearly found that the initial decomposition temperature (T-_5%_, temperature at mass loss of 5%) and maximum decomposition temperature (T_max_, temperature at maximum mass loss) of TPU composites are lower than those of pure TPU, indicating that the incorporation of MoS_2_ and LDH leads to the forward of the initial decomposition temperature, which is consistent with the results of CCT analysis. In addition, compared with the single MoS_2_ or LDH filled TPU and pure TPU, the LDH/MoS_2_ hybrid materials filled TPU have higher char yield. At 800 °C, the char yield of TPU/NiFeCe-LDH/MoS_2_ and TPU/NiFeTb-LDH/MoS_2_ reach up to 10.11% and 13.74%, respectively, attributing to the better catalytic carbonization of hybrid materials, and the carbon layer can protect the unburned TPU matrix [22].

It is easy to see from the DTG curves that TPU/LDH composites have a similar thermal decomposition process to TPU/LDH/MoS_2_. This can be explained by the low content of MoS_2_ in the TPU/LDH/MoS_2_, which makes MoS_2_ unable to efficiently participate in the carbonization process of TPU.

### 3.4. Char Residues Analysis of TPU Composites

To profoundly understand the flame retardant mechanism in the condensed phase, the carbon residues of TPU composites were further analyzed. Figure S4 gives the digital photos of char residues. Figure S4a displays the exposed aluminum foil, and the pure TPU is completely burned. In Figure S4b, TPU/MoS_2_ presents incomplete and fragile carbon residues after combustion, which also leads to the unsatisfactory flame retardancy and smoke suppression effects of the TPU. Although the carbon residues of TPU/LDH after combustion cover the entire aluminum foil, the carbon residues are relatively loose and there are hollows in the middle of the carbon residues. With the addition of LDH/MoS_2_ hybrid materials, the carbon residues of TPU/LDH/MoS_2_ are more complete and denser and the number of carbon residues increases significantly, which indicates that the LDH/MoS_2_ hybrid materials have a better catalytic carbonization effect.

**Figure 11:**
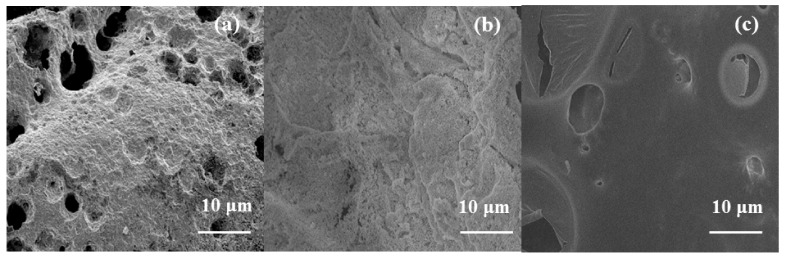
SEM images of carbon residues of TPU/MoS_2_ (**a**), TPU/NiFeTb-LDH (**b**) and TPU/NiFeTb-LDH/MoS_2_ (**c**).

The microstructure of carbon residues was further studied by SEM, as shown in Figure 11. It can be seen from Figure 11a that the carbon layer formed by TPU/MoS_2_ is discontinuous and the surface of the carbon layer is porous, which is not conducive to blocking heat, oxygen and combustible gases. For TPU added with NiFeTb-LDH, the morphology of carbon layer is relatively complete, but there are holes and cracks on the surface of the carbon layer. The holes can release the pyrolysis products from the underlying TPU matrix, thus reducing PHRR during combustion, but the reduction of THR is not obvious. As regards the carbon layer of TPU/ NiFeTb-LDH /MoS_2_, the carbon layer has fewer holes, compact appearance and smooth surface. The dense carbon layer can act as physical barrier to inhibit the further combustion of the TPU matrix, thereby improving the flame retardancy of the TPU [35].

**Figure 12:**
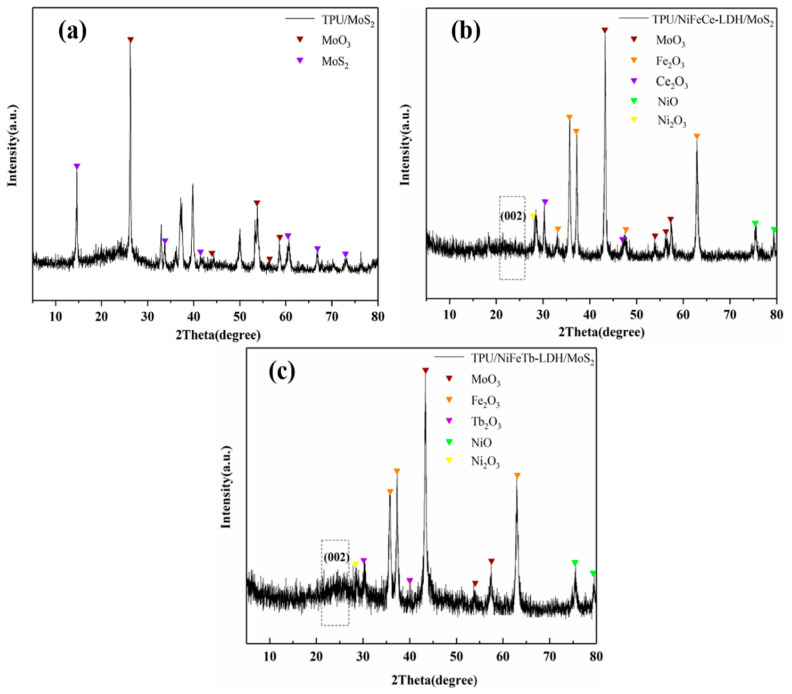
XRD patterns of carbon residues of TPU/MoS_2_ (**a**), TPU/NiFeCe-LDH/MoS_2_ (**b**) and TPU/NiFeTb-LDH/MoS_2_ (**c**).

The carbon residues of TPU composites were further studied by XRD, and the results are shown in Figure 12. As shown in Figure 12a, there are diffraction peaks of MoS_2_ and MoO_3_ in the XRD pattern of the carbon residues of the TPU/MoS_2_. However, the carbon residues of TPU/MoS_2_ have a low degree of graphitization, which also leads to a low carbon formation rate, poor flame retardancy and smoke suppression effects of TPU/MoS_2_ in the combustion process. For the XRD patterns of the carbon residues of TPU/NiFeCe-LDH/MoS_2_ (b) and TPU/NiFeTb-LDH/MoS_2_ (c), there are not only the diffraction peaks of MoO_3_ and graphite microcrystals, but also the diffraction peaks of metal oxides such as Fe_2_O_3_, NiO, Ni_2_O_3_ and Ce_2_O_3_/Tb_2_O_3_. The metal oxides help to form structurally stable carbon layers, which effectively limit the diffusion of oxygen and heat into the polymer interior.

### 3.5. Gas Analysis

Thermogravimetric analysis-infrared spectrometry (TG-FTIR) is an efficient approach for the dynamic analysis of gaseous products during combustion processes [36]. Figure S5 presents the 3D TG-FTIR spectra of pure TPU and TPU/NiFeTb-LDH/MoS_2_. As can be seen in Figure S5, the thermal decomposition process of TPU/NiFeTb-LDH/MoS_2_ is analogous to that of pure TPU, indicating that the addition of NiFeTb-LDH/MoS_2_ hybrid material does not change the pyrolysis products of TPU. The peaks of thermal decomposition products for TPU and TPU/NiFeTb-LDH/MoS_2_ are mainly distributed in the ranges of 3500–4000 cm^−1^, 2700–3000 cm^−1^, 2200–2500 cm^−1^, 1600–1800 cm^−1^ and 1300–1600 cm^−1^, which are consistent with previous literature [37].

**Figure 13:**
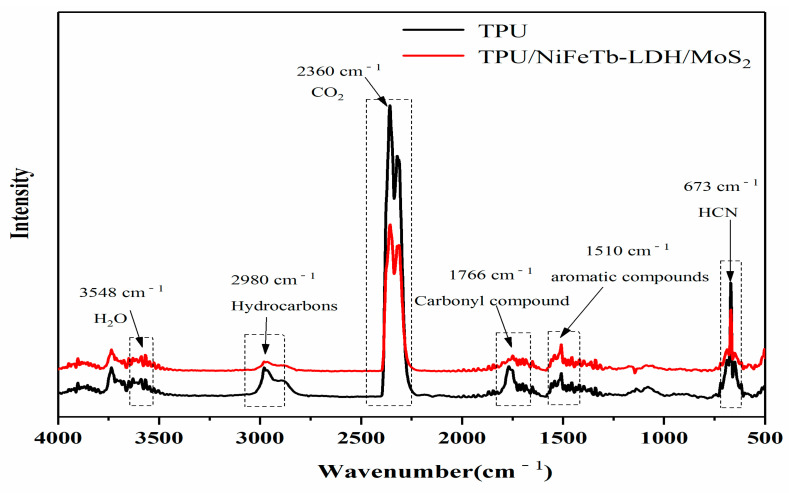
FTIR spectra of TPU and TPU/NiFeTb-LDH/MoS_2_ pyrolysis products at maximum decomposition rate.

Figure 13 presents the FTIR spectra of pure TPU and TPU/NiFeTb-LDH/MoS_2_ under the maximal decomposition rate. The absorption peak at 2980 cm^−1^ corresponds to the stretching vibration of the -CH_3_ or -CH_2_ group in hydrocarbons. The absorption peaks of HCN, CO_2_ and H_2_O are located at 673 cm^−1^, 2360 cm^−1^ and 3548 cm^−1^, respectively. In addition, the peaks at 1510 cm^−1^ and 1766 cm^−1^ correspond to the typical absorption peaks of aromatic hydrocarbons and carbonyl compounds, respectively.

**Figure 14:**
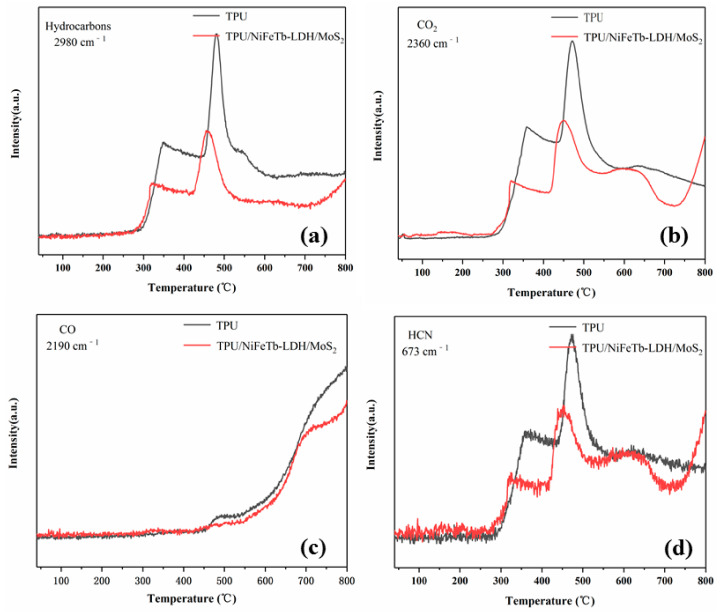
The absorbance intensities of Hydrocarbons (**a**), CO_2_ (**b**), CO (**c**) and HCN (**d**) of pure TPU and TPU/NiFeTb-LDH/MoS_2_.

In order to explicitly understand the difference of pyrolysis products between pure TPU and TPU/NiFeTb-LDH/MoS_2_, the main volatile pyrolysis products versus temperature were investigated, as depicted in Figure 14. It can be seen from Figure 14a that the absorbance intensity of the hydrocarbons of pure TPU is remarkably high compared to that of TPU/NiFeTb-LDH/MoS_2_. The decrement of hydrocarbons can further reduce the generation of smoke and increase fire scene visibility, which reduces fire rescue difficulties [38]. At the same time, the reduction of combustible hydrocarbons is beneficial to suppress HRR, which is consistent with the results of cone calorimetry. The CO and HCN are typical asphyxiating gases that can cause heavy casualties. In the presence of NiFeTb-LDH/MoS_2_, the release of CO and HCN from TPU is significantly lower than that of pure TPU, indicating that NiFeTb-LDH/MoS_2_ can effectively inhibit the release of toxic volatiles. In summary, NiFeTb-LDH/MoS_2_ endows TPU with better fire safety, which is the result of the catalytic effect of NiFeTb-LDH/MoS_2_ and the dense carbon layer as a physical barrier.

### 3.6. Flame Retardant Mechanism

**Figure 15:**
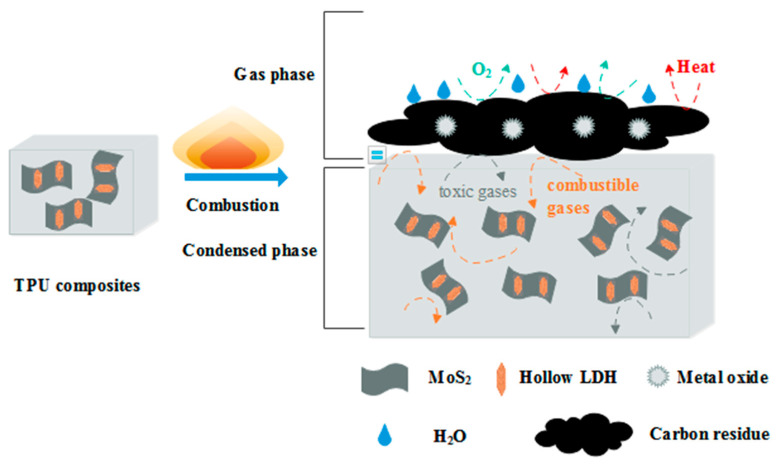
Illustration of the flame retardant mechanism of TPU/LDH/MoS_2_.

Combining the condensed phase-gas phase analysis and flame retardant performance results of TPU/LDH/MoS_2_, a rational flame retardant mechanism is proposed, as shown in Figure 15. The flame retardant mechanism of TPU composites in the condensed phase can be summarized as the following points: (1) The MoS_2_ nanosheets act as physical barriers during the combustion process of TPU, inhibiting the release of flammable and toxic gases from the underlying TPU composite. At the same time, MoO_3_ generated by the oxidation of MoS_2_ has a high-efficiency smoke suppression effect. (2) In the combustion process, the metal oxides formed by LDH can catalyze the polymer to form a carbon layer and absorb part of flue gas [22]. (3) The LDH/MoS_2_ hybrid materials improve the degree of graphitization and compactness of the carbon residues, and the protective carbon layer can act as a barrier between the combustion zone and the substrate to protect the unburned substrate. The flame retardant mechanism of TPU composites in the gas phase is mainly attributed to the fact that the water vapor generated by the thermal decomposition of LDH can effectively dilute flammable gases such as oxygen. Overall, the addition of LDH/MoS_2_ hybrid materials enhances the flame retardancy of TPU.

## 4. Conclusions

In this work, the novel 3D hollow LDH/MoS_2_ hybrid materials were synthesized by hydrothermal method and their composition and structure were characterized by XRD, FTIR, SEM, TEM and TGA. Afterward, the MoS_2_, LDH and LDH/MoS_2_ filled TPU composites were prepared by the melt blending method. The CCT results indicated that the flame retardancy and smoke suppression performance of TPU composites were greatly enhanced by the addition of LDH/MoS_2_ hybrid materials, and the PHRR and PSPR values were significantly reduced. The TGA results showed that the incorporation of LDH/MoS_2_ hybrid materials enhances the thermal stability of TPU composites. In addition, the existence of LDH/MoS_2_ hybrid materials inhibited the release of combustible volatiles (hydrocarbons) and the precipitation of toxic volatiles (CO and HCN) in TPU, indicating that LDH/MoS_2_ hybrid materials can dramatically enhance the fire safety of TPU. SEM and XRD results indicated that the metal oxides generated during the combustion of TPU composites contribute to compact carbon layers, and the protective carbon layers can act as barriers between the combustion zone and the matrix to protect the unburned matrix. To sum up, the improvement of flame retardancy and smoke suppression performance, thermal stability and fire safety of TPU/LDH/MoS_2_ can be ascribed to the catalytic carbonization of LDH as well as the physical barrier effect of MoS_2_.

## Figures and Tables

**Table 1:** Cone calorimeter data of TPU composites.

Sample Code	PHRR kW/m^2^	THR MJ/m^2^	PSPR m^2^/s	TSP m^2^
TPU	1135	118.8	0.113	12.3
TPU/MoS_2_	804	104.6	0.103	11.9
TPU/NiFeCe-LDH	710	108.5	0.065	8.1
TPU/NiFeTb-LDH	652	114.6	0.049	9.5
TPU/NiFeCe-LDH/MoS_2_	581	98.4	0.050	7.4
TPU/NiFeTb-LDH/MoS_2_	557	97.2	0.045	7.9

**Table 2:** Thermogravimetry data of TPU composites.

Sample Code	T-_5%_ (°C)	T_max_ (°C)	Char Yield (%)
TPU	333	454	5.85
TPU/MoS_2_	317	432	7.93
TPU/NiFeCe-LDH	302	385	7.58
TPU/NiFeTb-LDH	308	382	9.05
TPU/NiFeCe-LDH/MoS_2_	316	376	10.11
TPU/NiFeTb-LDH/MoS_2_	316	394	13.74

## Supplementary Materials

The following supporting information can be downloaded at: https://www.mdpi.com/article/10.3390/polym14081506/s1, Figure S1: Plan scan images and EDS spectrum of NiFeCe-LDH/MoS_2_ (**a,b**) and NiFeTb-LDH/MoS_2_ (**c**,**d**); Figure S2: N_2_ adsorption-desorption isotherms of NiFeCe-LDH (**a**) and NiFeTb-LDH (**b**); Figure S3: XPS spectra of NiFeCe-LDH/MoS_2_ (**a**) and NiFeTb-LDH/MoS_2_ (**b**); Figure S4: Digital photos of carbon residues of TPU (**a**), TPU/MoS_2_ (**b**), TPU/NiFeCe-LDH (**c**), TPU/NiFeTb-LDH (**d**), TPU/NiFeCe-LDH/MoS_2_ (**e**) and TPU/NiFeTb-LDH/MoS_2_ (**f**); Figure S5: TG-FTIR spectra of thermal decomposition products of TPU (**a**) and TPU/NiFeTb-LDH/MoS_2_ (**b**).
