# Supplementary material for: Synthesis of 3D Hollow Layered Double Hydroxide-Molybdenum Disulfide Hybrid Materials and Their Application in Flame Retardant Thermoplastic Polyurethane
Source: Polymers (Basel). 2022 Apr 7;14(8):1506. doi: 10.3390/polym14081506 (PMC9029036; doi:10.3390/polym14081506)
Supplement: Supplementary file 1 [file polymers-14-01506-s001.zip › polymers-1659620-supplementary.pdf]

polymers

MDPI

Supplementary Materials: Synthesis of 3D Hollow Layered Double Hydroxide-Molybdenum Disulfide Hybrid Materials and Their Application in Flame Retardant Thermoplastic Polyurethane

Yi Qian, Wenyuan Su, Long Li, Haoyan Fu, Jiayin Li and Yihao Zhang

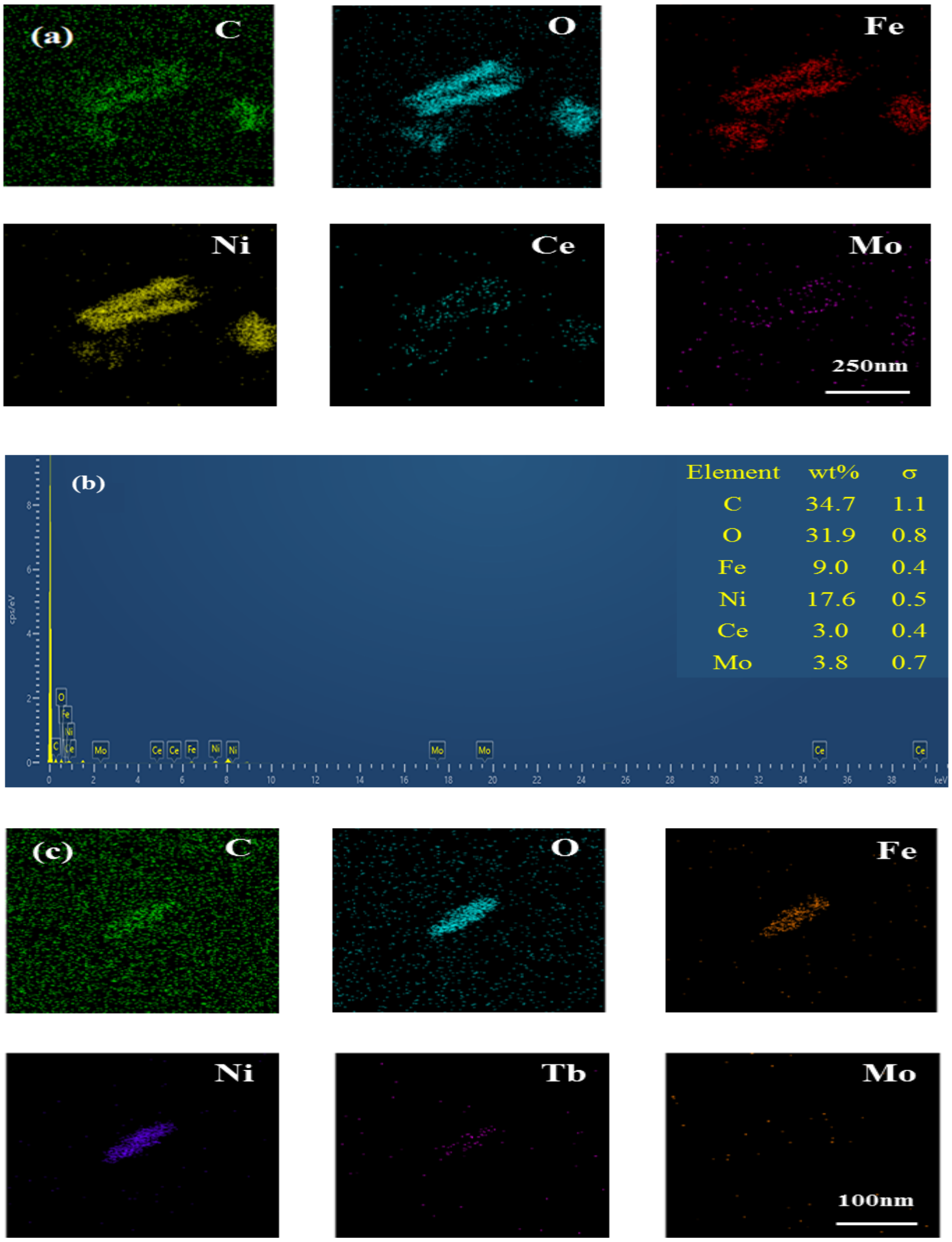

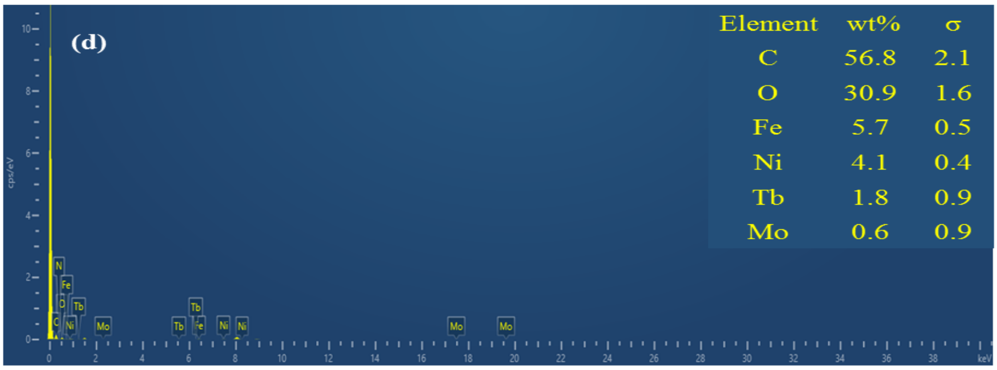

Figure S1. Plan scan image and EDS spectrum of NiFeCe-LDH/MoS_2 (**a**,**b**) and NiFeTb-LDH/MoS_2 (**c**,**d**).

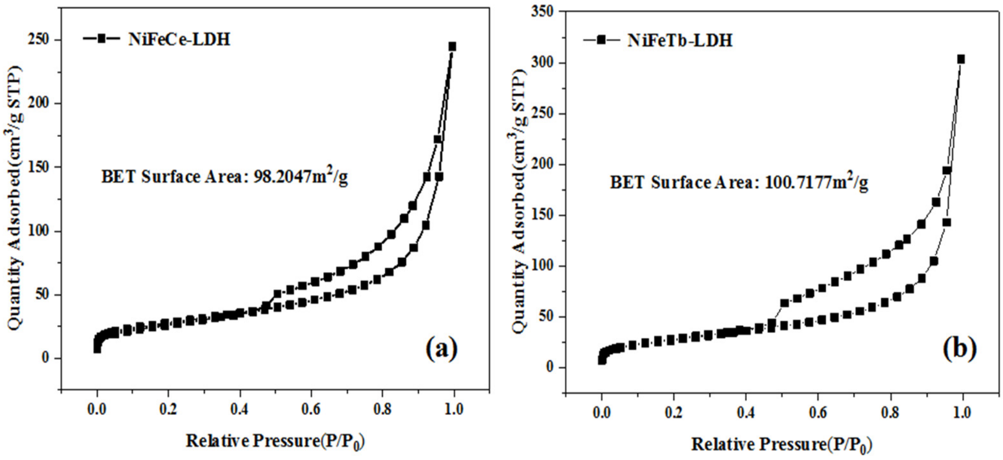

Figure S2. N_2 adsorption-desorption isotherms of NiFeCe-LDH (**a**)and NiFeTb-LDH (**b**).

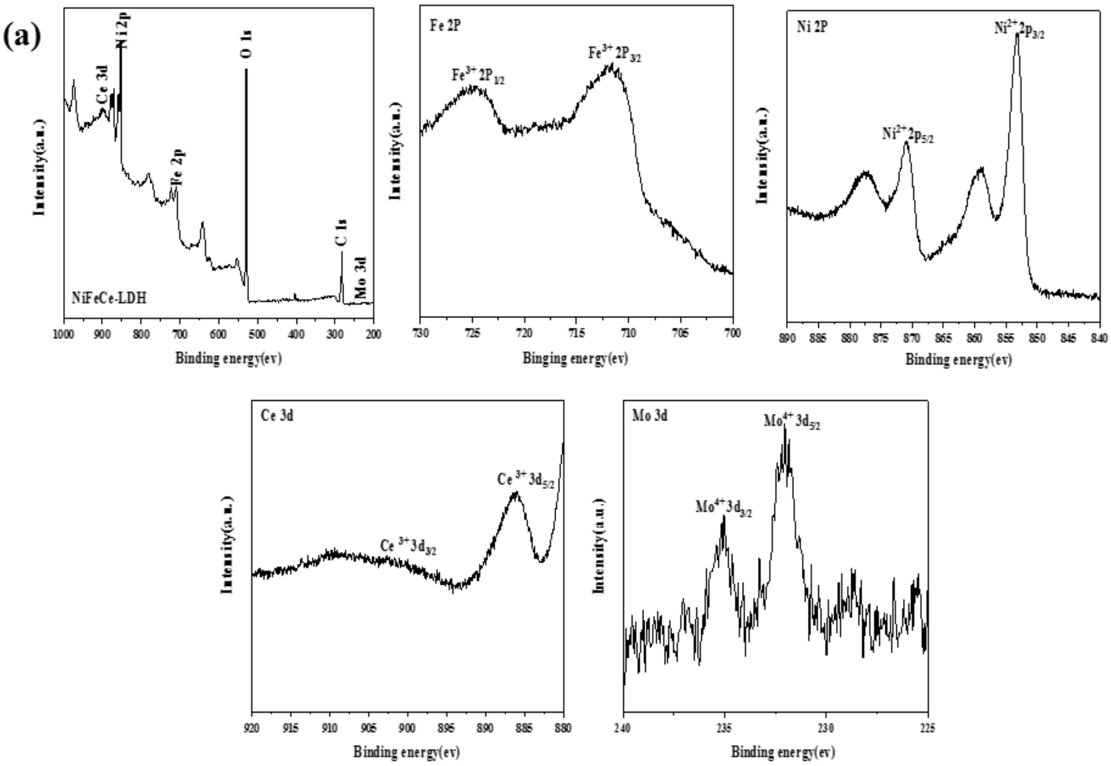

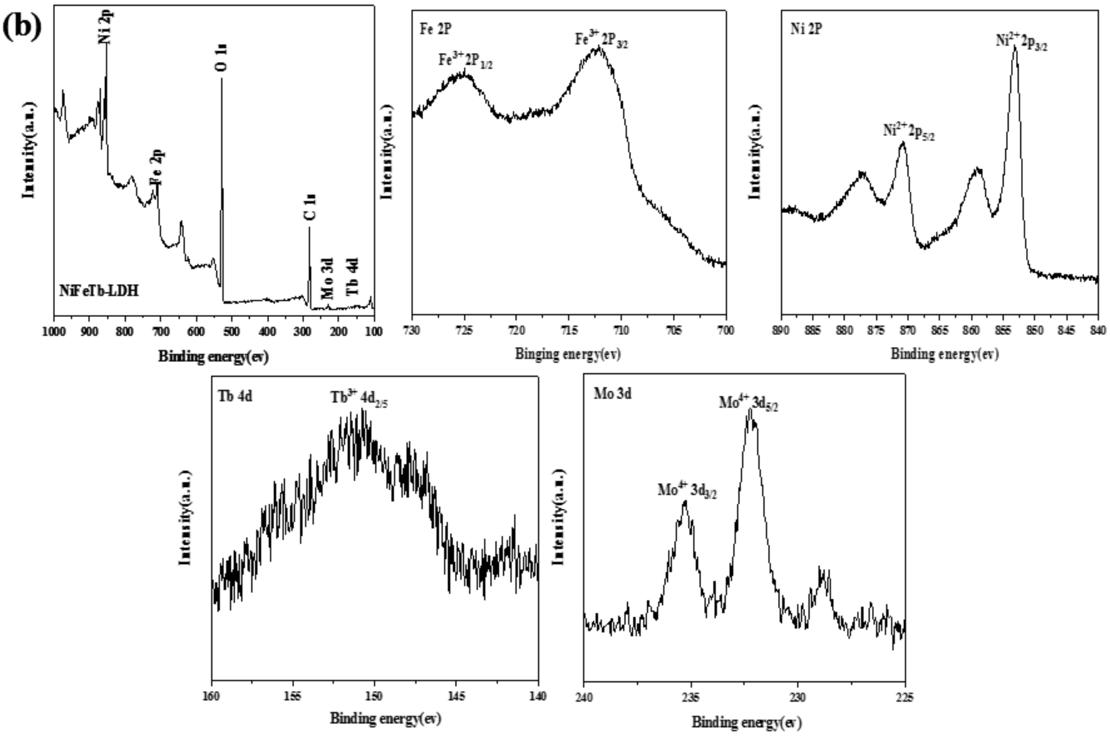

Figure S3. XPS spectra of NiFeCe-LDH/MoS_2 (**a**)and NiFeTb-LDH/MoS_2 (**b**).

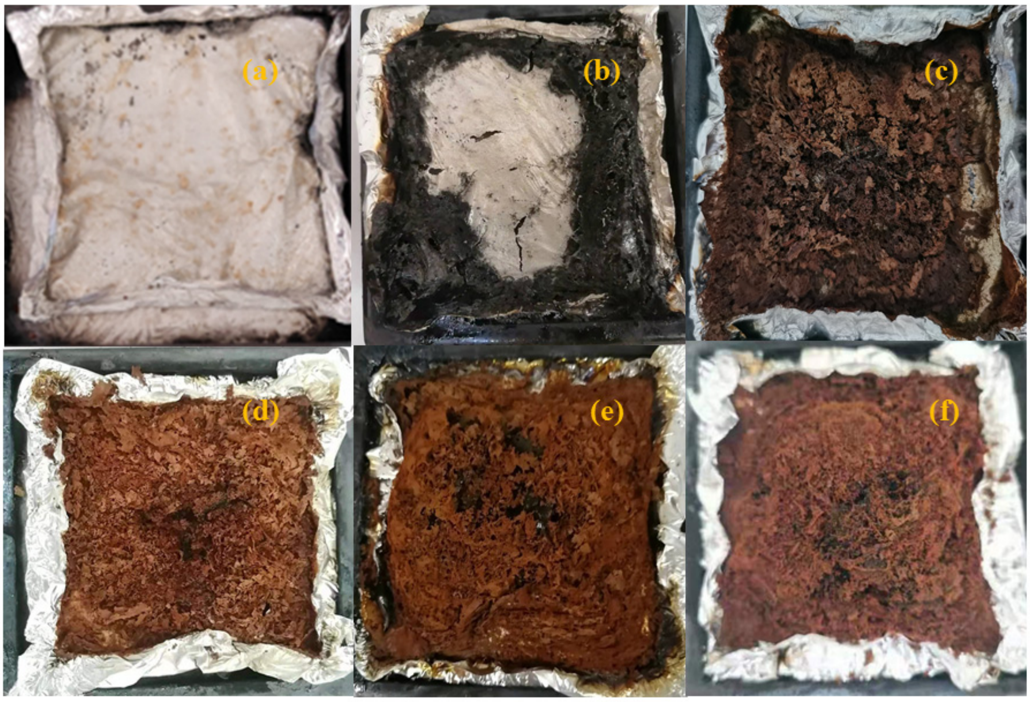

Figure S4. Digital photos of carbon residues of TPU (**a**), TPU/MoS_2 (**b**), TPU/NiFeCe-LDH (**c**), TPU/NiFeTb-LDH (**d**), TPU/NiFeCe-LDH/MoS_2 (**e**), TPU/NiFeTb-LDH/MoS_2 (**f**).

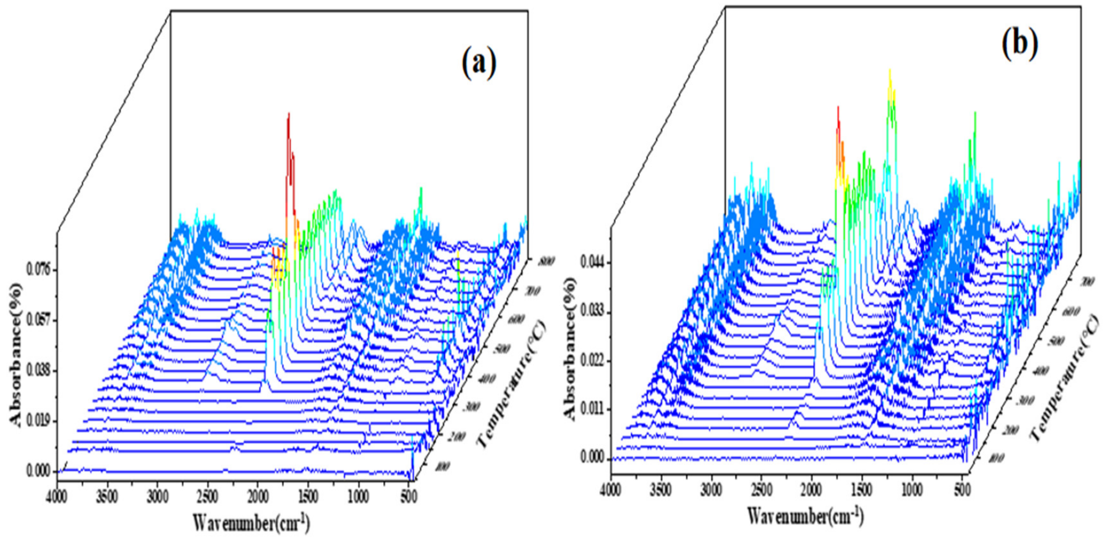

Figure S5. TG-FTIR spectra of thermal decomposition products of TPU (**a**) and TPU/NiFeTb-LDH/MoS_2 (**b**).
